# Pet imaging of cytotoxic human T cells using an 89Zr-labeled anti-CD8 minibody

**DOI:** 10.1186/2051-1426-3-S2-P388

**Published:** 2015-11-04

**Authors:** Tove Olafsen, Michael Torgov, Green G Zhang, Jason Romero, Charles Zampila, Filippo Marchioni, Karen Jiang, Jean Gudas, Daulet Satpayev

**Affiliations:** 1 ImaginAb, Inc., Inglewood, CA, USA

## Background

Major challenges to advancing the new wave of cancer immunotherapies are to select patients who will respond to single immunotherapies, identify those who need more tailored combination regimens, and then, determine early during treatment whether the therapy is working. “ImmunoPET” imaging of tumor infiltrating T cells with radiolabeled antibody fragments can provide a specific and sensitive modality to achieve this goal. For this purpose we have developed ^89^Zr-Df-IAB22M2C, an anti-CD8 minibody (Mb) conjugated with desferrioxamine (Df) and radiolabeled with ^89^Zr for imaging human CD8^+^T cells in man.

## Methods

Df-isothiocyanate was conjugated to IAB22M2C on lysine residues and the biologic activity assessed using a panel of functional and biochemical assays. After radiolabeling with ^89^Zr, pharmacokinetic, biodistribution and PET imaging studies were performed in NOD scid gamma (NOD.Cg-Prkdc^scid^ Il2rg^tm1Wjl^/SzJ; NSG) mice engrafted with human peripheral blood mononuclear cells (PBMCs) or CD34^+^ stem cells.

## Results

Conjugation of IAB22M2C with Df chelators did not impact structural integrity or binding to CD8 (EC_50_ 1.2 nM). A panel of i*n vitro* functional assays using donor PBMCs demonstrated that Df-IAB22M2C did not activate or stimulate T cells to proliferate. Importantly, Df-IAB22M2C did not deplete or activate human T cells in NSG mice engrafted with human CD34^+^ stem cells. NSG mice engrafted with human PBMCs are reproducible models of xenogeneic Graft Versus Host Disease (GVHD). ^89^Zr-Df-IAB22M2C, radiolabeled to a specific activity of ~30-40 µCi/µg, was able to detect CD8^+^T in the spleens of mice one week post PBMC engraftment. As GVHD progressed 4-5 weeks later, expansion and trafficking of the engrafted T cells to extra-lymphoid tissues including lungs could be followed. Terminal biodistribution showed a 2-3 fold increase in radioactivity uptake in lungs (from 4 to 9.2 % ID/gm) at 1 and 4 weeks respectively; a result that was confirmed by IHC analysis. ^89^Zr-Df-IAB22M2C clearance followed a biphasic distribution with a terminal half-life of approximately 2.7 hrs indicating that same day imaging is likely in humans. These results are being extended to include human tumor models implanted in humanized mice.

## Conclusion

^89^Zr-Df-IAB22M2C radiolabeled to high specific activity can be used to track T cell trafficking *in vivo* and will provide a sensitive means for monitoring CD8^+^T cell distribution in patients treated with different immunotherapies. The safety profile and biochemical properties of ^89^Zr-Df-IAB22M2C *in vitro* and *in vivo* support the commencement of human clinical trials with this probe early next year.

